# Biofilm Formation, Modulation, and Transcriptomic Regulation Under Stress Conditions in *Halomicronema* sp.

**DOI:** 10.3390/ijms26020673

**Published:** 2025-01-15

**Authors:** Marina Caldara, Henk Bolhuis, Marta Marmiroli, Nelson Marmiroli

**Affiliations:** 1 Department of Chemistry, Life Sciences and Environmental Sustainability, University of Parma, Parco Area delle Scienze 11/A, 43124 Parma, Italy; marta.marmiroli@unipr.it (M.M.); nelson.marmiroli@unipr.it (N.M.); 2 Interdepartmental Center SITEIA.PARMA, University of Parma, Parco Area delle Scienze 33, 43124 Parma, Italy; 3 Department of Marine Microbiology and Biogeochemistry (MMB), NIOZ Royal Netherlands Institute for Sea Research, Landsdiep 4, 1797 SZ Den Hoorn, Texel, The Netherlands; henk.bolhuis@nioz.nl

**Keywords:** cyanobacteria, biofilm formation, stress resilience, transcriptomics, EPS, *Halomicronema* sp.

## Abstract

In nature, bacteria often form heterogeneous communities enclosed in a complex matrix known as biofilms. This extracellular matrix, produced by the microorganisms themselves, serves as the first barrier between the cells and the environment. It is composed mainly of water, extracellular polymeric substances (EPS), lipids, proteins, and DNA. Cyanobacteria form biofilms and have unique characteristics such as oxygenic photosynthesis, nitrogen fixation, excellent adaptability to various abiotic stress conditions, and the ability to secrete a variety of metabolites and hormones. This work focused on the characterization of the cyanobacterium *Halomicronema* sp. strain isolated from a brackish environment. This study included microscopic imaging, determination of phenolic content and antioxidant capacity, identification of chemicals interfering with biofilm formation, and transcriptomic analysis by RNA sequencing and real-time PCR. Gene expression analysis was centered on genes related to the production of EPS and biofilm-related transcription factors. This study led to the identification of *wza1* and *wzt* as EPS biomarkers and *luxR-05665,* along with genes belonging to the TetR/AcrR and LysR families, as potential biomarkers useful for studying and monitoring biofilm formation under different environmental conditions. Moreover, this work revealed that *Halomicronema* sp. can grow even in the presence of strong abiotic stresses, such as high salt, and has good antioxidant properties.

## 1. Introduction

Cyanobacteria are Gram-negative microbes widespread in numerous habitats, such as terrestrial, freshwater, and hypersaline environments [1]. All species belonging to this phylum are mostly obligate oxygenic photoautotrophs, capable of using carbon dioxide (CO_2_) as their main carbon source and light as their main energy source [2]. To support these functions, they contain phycobiliproteins, which act as accessory pigments in photosynthesis [3].

Due to their inherent ability to fix nitrogen and CO_2_ and their excellent adaptability to various abiotic stress conditions [4], cyanobacteria can be considered excellent biofertilizers since they improve soil fertility and texture, resulting in increased crop productivity. Their ability to release antimicrobials makes them ideal candidates for use as biocontrol agents and as an alternative to chemical pesticides [5]. Moreover, some cyanobacteria can detoxify or degrade various pollutants, both natural and xenobiotic, and can uptake heavy metals through the use of metallothioneins [6,7]. In addition, cyanobacteria play an important role in rhizospheric engineering [8]. These organisms are capable of secreting biologically active compounds such as phytohormones, amino acids, sugars, phenols, and vitamins, which act as signaling molecules for the synthesis of a series of plant secondary compounds [9]. Biocrusts are cyanobacteria-dominated biofilms that develop on the soil surface and comprise a complex community of other microorganisms. These biocrusts improve water-holding capacity, increase infiltration, and decrease erosion while maintaining soil moisture for a longer period [4,10,11]. The cyanobacteria are largely responsible for the formation of extracellular polymeric substances (EPS), and their composition varies according to the species, nutrient availability, growth stage, and environmental conditions [12,13,14,15]. Such substances consist mainly of polysaccharides and may remain associated with the cell surface or be released into the surrounding environment [15]. Besides playing a fundamental role in cell aggregation and adhesion, they can perform different functions, including protection from desiccation, viral attack, UV radiation, or sequestration of nutrients and metal ions [16,17].

Knowledge of EPS production in cyanobacteria is limited. The synthesis typically starts in the cytoplasm, where monosaccharides are converted into sugar nucleotides. These are then transferred by specific glycosyltransferases to transporter molecules in the plasma membrane. The subsequent steps of polymerization, assembly, and export are usually fairly conserved in bacteria [18]. Three main mechanisms have been described: the Wzy-dependent pathway, the ABC-dependent transporter pathway, and the synthase-dependent pathway [19]. Analysis at the phylum level shows that most cyanobacteria carry genes encoding proteins involved in one of the three pathways, but often not the full set that characterizes it. Moreover, EPS may be located loosely outside the cells (soluble fraction) or, in flocs or biofilms, closely associated with the cells [20]. Other important elements of EPS are lipopolysaccharides (LPS), which also play a key role in the stabilization of biocrusts [4,10]. Lipopolysaccharides are large and complex amphipathic molecules consisting of lipids and saccharide residues that constitute the outer leaflet of the cell membrane of Gram-negative bacteria [21].

*Halomicronema* sp. is a cyanobacterium found in brackish water and microbial mats, where it forms biofilms. In this study, the isolate *Halomicronema* sp. CCY15110 was considered; this strain was isolated from coastal microbial mats on the North Sea beach of Schiermonnikoog Island in the Netherlands [22] and its genome has been sequenced [23]. This study aimed to better characterize the biofilm formation of this bacteria, to identify chemicals that can inhibit this process, and to study gene expression regulating biofilm formation, including EPS genes.

## 2. Results and Discussion

### 2.1. Growth of Halomicronema sp. and Biofilm Formation

**Figure 1:**
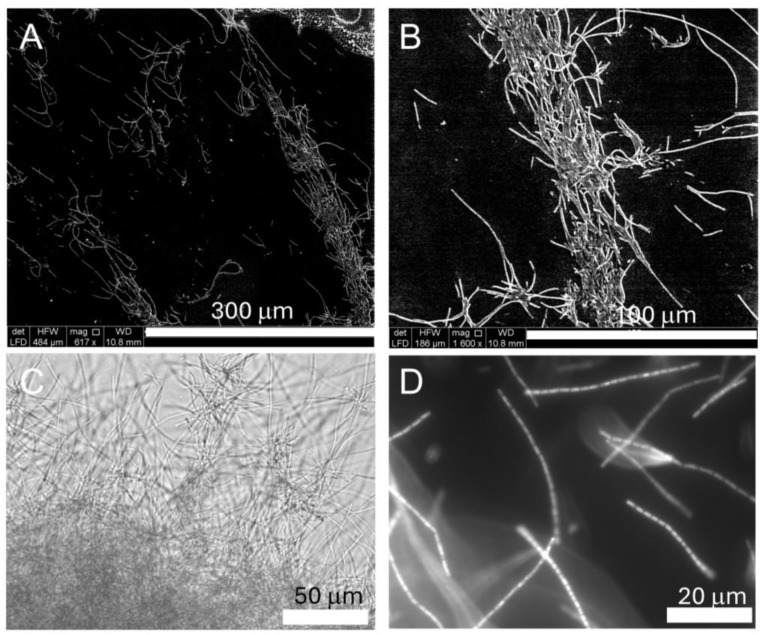
Imaging *Halomicronema* sp. with ESEM in low vacuum mode (**A**,**B**), light microscope (**C**), and with fluorescence microscopy after staining with SYTO-9 (**D**); for image (**C**), the magnification used was 40×, and for image (**D**), the magnification was 100×. For each image, the scale bar is reported at the bottom right.

For imaging biofilm formation, cultures of *Halomicronema* sp. were grown on slides. ESEM images in low vacuum mode (Figure 1A,B) showed that *Halomicronema* sp. forms long tangled filaments, a characteristic that was also observed with classical light microscopy (Figure 1C). Using fluorescence staining with SYTO-9, which stains DNA, it was possible to visualize individual cells within these filaments (Figure 1D).

**Figure 2:**
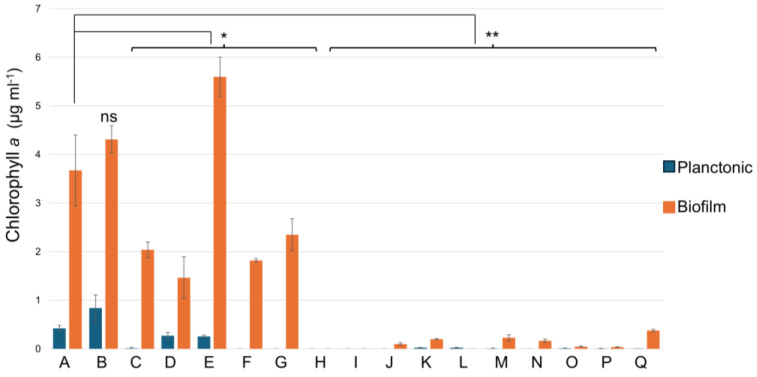
Biomass formation of cyanobacteria planktonic and biofilm fractions detected through chlorophyll measurements. *Halomicronema* sp was grown in BA+ medium for 7 days under 16 h light and 8 h dark; temperature was constant at 23 °C for all the conditions tested. The strain was also tested in the presence of different chemicals: A = CTR the control condition, B = NaCl 340 mM, C = NaCl 680 mM, D = NaCl 1 M, E = MgSO_4_ (50 mM), F = MgCl_2_ (250 mM), G = EDTA (250 µM), H = CaCl_2_ (500 mM), I = Glucose 110 mM (2%), J = KH_2_PO_4_ (125 mM), K = CaCl_2_ (250 mM), L = EDTA (2.5 mM), M = Na-acetate (50 mM), N = Na-citrate (50 mM), O = (NH_4_)_2_HPO_4_ (100 mM), P = (NH_4_)HSO_4_ (100 mM), Q = KH_2_PO_4_ (50 mM). The data represent the mean and the standard deviation of three independent biological replicates. A Student’s *t*-test was performed to compare the effects of the different chemicals. The effects were all significant, except for NaCl 340 mM (* was used for *p*-values < 0.05 and ** for *p*-values < 0.01; ns: not significant).

Next, cyanobacteria were grown in a liquid BA+ medium in 24-well plates. Here, cells of *Halomicronema* sp. grew predominantly in a biofilm attached to the bottom of the wells with few planktonic cells in the supernatant (Figure 2 condition A). This cyanobacterium is relatively insensitive to the highest concentration of salt tested; even with 1 M NaCl, it displays almost half the biomass with respect to the control condition (Figure 2, condition D). In some cyanobacteria, salt stress increases EPS production, a phenomenon that was suggested to be linked to a higher salt tolerance [24]. Higher tolerance to salt is a phenotype not present in cyanobacteria with a thick and firmly attached capsule [17]. The presence of any of the other tested chemicals disturbed its growth, except for MgSO_4_, which induces biofilm formation (Figure 2, condition E). The effect could be the result of both the presence of sulfate and Mg^2+^. Indeed, sulfur is an essential nutrient and is needed to support different functions such as photosynthesis, respiration, and redox metabolism [25], while Mg^2+^, as well as Ca^2+^, forms ion bridging that strengthens the EPS matrix [26,27]. Not surprisingly, the chelating agent EDTA at a 250 µM concentration did not have a strong influence, but at 2.5 mM, it completely inhibited growth (Figure 2, conditions G and L). EDTA destabilizes biofilms by sequestering calcium, iron, magnesium, and zinc [28].

### 2.2. Phenolic Content and Antioxidant Capacity

Phenolic compounds containing one or more hydroxyl groups (such as flavonoids, gallic acid, tannins, and hydroxybenzoic acid) are able to chelate metals, decompose hydrogen peroxide, and have the capacity to act as antioxidants. The total phenolic content, (0.7 mg GAE g^−1^, where GAE is Gallic Acid Equivalent, see Table 1) fell between previously reported values in other cyanobacteria (0.54 mg GAE g^−1^ extract measured in *Merismopedia* sp. and 2.45 mg GAE g^−1^ extract found in *Synechocystis salina*) [29,30]. The phenolic content was reported to be slightly positively correlated with the DPPH (1,1-diphenyl-2-picrylhydrazil) inhibition assay and the ABTS (2,2-azino-bis-3-ethylbenzothiazoline-6-sulphonic acid) antioxidant test [29]. The antioxidant capacity in these assays was high when compared to isolates of *Arthrospira platensis*, *Lithothamnium calcareum*, *Synechocystis salina*, *Cyanobium* sp., *Tychonema* sp., and similar to *Nodosilinea nodulosa* [30,31]. Overall, the data suggest that *Halimicronema* sp. has good antioxidant potential (Table 1).

EPS content was measured previously [32]. The results showed that soluble EPS ranged, depending on the growing conditions, between 47 and 188 mg L^−1^, while for the bound EPS, the range was 38–211 mg L^−1^. Similarly, the protein fraction of the EPS reached 2.4–12.4 mg L^−1^ within the soluble EPS and could go up to 72–100 mg L^−1^ within the bound EPS [32].

### 2.3. Expression Analysis of EPS-Related Genes

The EPS structure is a key element of a biofilm. After having listed all the components involved in the three main bacterial pathways of EPS assembly and export (Wzy-dependent, ABC transporter-dependent, and synthase-dependent) and those responsible for lipopolysaccharide export [10,17,19], their presence in the genome of *Halomicronema* sp. was verified using NCBI and txid 2767773 ([23], https://www.ncbi.nlm.nih.gov/bioproject/?term=PRJNA658956, accessed on 10 July 2023). We focused on the last stages of EPS biosynthesis as they are the best-known and most conserved among the various bacterial species. Table S3 shows the proteins that were identified in *Halomicronema* sp. As can be seen, most of the genes involved in these mechanisms encode for proteins characteristic of the Wzy-dependent pathway, suggesting that in *Halomicronema* sp., the assembly and export of EPS most often proceed through this system, but the complete assembly pathway is not present. The same can be observed for the lipopolysaccharides export pathway.

Halotolerance in cyanobacteria has been reviewed recently [33,34], while salt acclimation in *Synechocystis* sp. has been reviewed previously [35]. Data from *Synechocystis* sp showed that many of the factors involved in this response, such as the expression of the *gpp*S gene and the accumulation of glucosylglycerol [36], the sensory histidine kinases that transduce salt stress signals [37], and heat shock proteins [38]. In *Synechocystis* sp., different stresses trigger similar stress-related genes [38]. In all these studies, information on EPS-related genes is missing.

**Figure 3:**
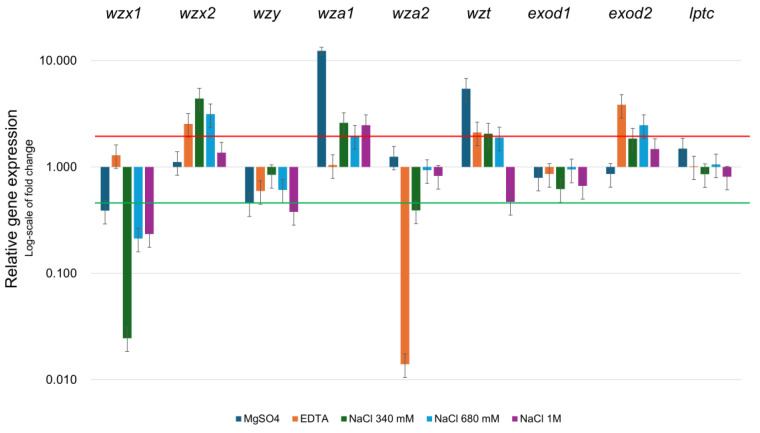
Gene expression analysis of EPS-related genes from *Halomicronema* sp, cells were grown in six different growing conditions: control, and in the presence of EDTA 250 µM, or MgSO_4_ 50 mM, NaCl 340 mM, NaCl 680 mM, and NaCl 1 M; expression is relative to the control condition using the 2^−ΔΔ^Ct method. Red line represents a 2 logfold increased expression, while the green line represents a 2 log-fold decrease in expression. Data outside these two boundaries were considered significant. The data represent the mean and the standard deviation of three independent biological replicates.

Gene expression of the identified EPS genes (Figure 3) was sensitive to the conditions tested. Specifically, *wzx* genes, involved in transferring the lipid-bound oligosaccharide repeat units to the periplasmic side of the plasma membrane [19,39], displayed opposite behaviors: *wzx*2 was overexpressed in the conditions tested, while *wzx*1 was repressed. *wzy*, a gene that polymerases polysaccharides units, is mildly downregulated in all the conditions tested. *wza*1, which exports polymer across the outer membrane [18,19], was over-expressed in most of the conditions tested except in the presence of EDTA, while *wza*2 was repressed by EDTA and at the lowest NaCl concentration. *wzt*, which transfers the polysaccharide that has been fully polymerized to the inner leaflet of the plasma membrane [19], was slightly induced, except at the highest concentrations of NaCl. *exod*1, whose function is still not clear, was over-expressed in all the conditions except when MgSO_4_ was present in the growing medium. *wzy*, which polymerizes polysaccharide units, was repressed when NaCl or EDTA were present. In the previous section, it was reported that MgSO_4_ was the only chemical that stimulated biofilm formation. This phenotype is supported also by gene expression. Specifically, Figure 3 shows that *wza2* and *exod2* have opposite expression profiles when MgSO_4_ is present in the medium or when any of the other chemicals. Moreover, the expression of *wza1* and *wzt* is much higher when MgSO4 is present compared to the other conditions. All the genes studied were modulated except for *exod1* and *lptc*, which maintained constant expression levels. This genetic distribution in *Halomicronema* sp. seems similar to what has been described in *Synechocystis* sp. strain PCC 6803 [40]; however, in *Halimicronema* sp., the *kpsM* gene was not identified. This gene was reported as the only EPS-related gene that, when mutated, impairs the secretion of EPS [40].

### 2.4. Transcriptomic Profile of Halomicronema sp.

Next, to know more about the general gene expression profile of *Halomicronema* sp., an RNA sequencing experiment was performed on the strain grown under control conditions. Here, about 20 million reads were obtained for each biological replicate with an average input read length of 292 bp and a mean quality score of 36 (Table S1). More than 76% of the reads aligned with the reference genome of *Halomicronema* sp. CCY15110. More specifically, a total of 5492 different genes were detected, of which 1268 (23%) encode for proteins with unknown function (or defined as “hypothetical proteins”). The most abundant transcript (Table S2), which had a value of 1,283,853 reads, is that for transfer-messenger RNA (tmRNA or SsrA) (values were normalized for gene length and total reads abundance per sample). The tmRNA is a bacterial RNA molecule that functions as both transfer and messenger RNA. In fact, it possesses paired and structured ends similar to a tRNA molecule, capable of loading alanine, while the central sequence of the molecule contains a short ORF coding for about ten amino acids, including various alanines, and ends with a stop codon [41]. It forms a ribonucleoprotein complex (tmRNP) along with Small Protein B (SmpB), elongation factor EF-Tu, and ribosomal protein S1. The key role of tmRNA is to bind stalled ribosomes and add a tag to the C-terminus of the unfinished polypeptide, inducing its proteolysis. This process assures translational quality and optimal regulation of gene expression. This process is known as a trans-translation reaction [42]. Given its importance, tmRNA is conserved and it is one of the most abundant RNAs in all bacteria [43]. As expected, analyzing the most commonly present transcripts (at least 5000 reads) showed that they code for proteins playing a role in photosynthesis (photosystem I and II related proteins, cytochrome), and among them, there are also genes involved in the synthesis of phycobilisome, phycocyanin, and allophycocyanin. These proteins form elaborate antennae capturing sunlight and transferring the energy to the photosynthetic reaction systems [44]. These protein complexes are located on the thylakoid and exhibit different abundance during their biogenesis and under light-activated heterotrophic growth [45,46]. Genes coding for transporters of iron, phosphate, and ammonium are also highly represented (Table S2). This is not surprising since it is known that the availability of those components can be a bottleneck for the growth of cyanobacteria in various ecosystems [47,48,49]. Interestingly, growth as entangled filaments (a phenotype seen in *Halomicronema* sp., see Figure 1) in *Trichodesmium* has been linked to an enhanced capacity to acquire iron from particulate sources such as dust, a physical structure that can facilitate the growth of a complex bacterial community [49,50]. Similarly, it is possible that *Halomicronema* sp. plays a similar role in the microbial mat where it was isolated. Finally, among the most abundant transcripts, we find genes coding for regulators of protein synthesis (sigma factors, elongation factors, and ribosomal proteins), other transcripts are linked to the production of ABC transporters and metalloproteases. In addition, there are numerous transcripts coding for transcription factors, transposases, methyltransferases, and various transporters. Considering the important regulatory work that transcription factors carry out, including regulating biofilm formation, the next analysis was performed on a subset of these proteins.

### 2.5. Expression Analysis of Main Transcription Regulators

Next, nine families of transcription regulators were studied as they are involved in virulence, biofilm formation, and development (Table 2). For each family (Table 2), the biological role and a selection of transcription factors further analyzed for each category are reported, along with their Gene_id, average reads value (considering the RNA-seq experiment), the Protein_id, and the average read values found in the RNA seq experiment. For each of these genes, specific primers were designed, and their expression was studied by real-time quantitative PCR (RTqPCR) in the presence of five different biofilm modulators: 340 mM NaCl, 680 mM NaCl, 1 M NaCl, 50 mM MgSO_4_, and 250 µM EDTA.

**Figure 4:**
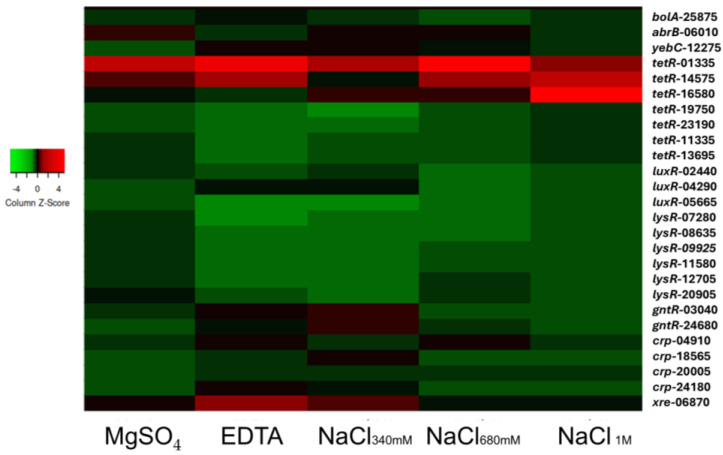
Gene expression analysis of selected transcriptional regulators from *Halomicronema* sp, cells were grown in six different growing conditions: control, and in the presence of EDTA 250 µM, or MgSO_4_ 50 mM, NaCl 340 mM, NaCl 680 mM, and NaCl 1 M. Expression is relative to the control condition using the 2^−ΔΔ^Ct method, and it represented with a heat-map obtained by using www.heatmapper.ca (accessed on 1 July 2024).

Factors belonging to the AbrB family and the YebC/PmpR family, which play an essential role in biofilm formation, show a lower expression level (Figure 4) when NaCl was added at the highest concentration [51,52]. TetR/AcrR regulates essential processes, including metabolism, virulence, resistance, and biofilm formation [53,54]. The selected genes belonging to the TetR/AcrR family exhibited opposite characteristics depending on the gene being considered (Figure 4); *tet*R-01335 and *tetR*-14575 were strongly overexpressed, while the expression of *tetR*-16580, *tetR*-19750, *tetR*-23190, *tetR*-11335, and *tetR*-13695 was repressed with respect to the control condition. All LuxR family genes, which play a key role in quorum sensing in Gram-negative bacteria, and regulate virulence, motility, and biofilm formation [55,56], were repressed. Similarly, those of the LysR, GntR, and Crp/Fnr protein families, all involved in virulence processes [57,58,59,60], were downregulated compared to the control. The XRE protein, which was suggested to be implicated in virulence [61], decreased with increasing NaCl concentration, remained nearly constant in the presence of 50 mM of MgSO_4_, and increased if 250 μM of EDTA was introduced into the culture medium. BolA factor, which regulates the transcription of several genes coding for proteins implicated in the transition between the planktonic stage and biofilm development, spore formation, and cell survival [62], was repressed in all the conditions tested when compared to the control. Differences in gene expression due to the presence of MgSO_4_ were displayed only for a few genes. Searching for the RNA-seq data for the EPS genes, only the expression of the gene *lptc* was clearly identified (average reads 2830). The other EPS-related genes were assigned general categories, such as the “ABC-related transporter”, and no clear correspondence was identified between this general category and the genes described within Section 2.3.

## 3. Materials and Methods

### 3.1. Growing Conditions

*Halomicronema* sp. (CCY15110) [22] was grown in 24-well plates in 1 mL of BA+ medium (https://www.dsmz.de/microorganisms/medium/pdf/DSMZ_Medium1677.pdf, accessed on 1 March 2023) per well at the constant temperature of 23 °C with a photoperiod of 16 h light and 8 h dark. The biomass was estimated by measuring the chlorophyll content [63,64]. Several chemicals have been reported to influence the formation of biofilms and EPS (i.e., nitrogen phosphate, sulfate, salt content, and light), as described in Pereira et al., 2015. The effects of different substances were tested by growing the bacteria in the presence of different growing conditions (340 mM NaCl, 680 mM NaCl, 1M NaCl, MgSO_4_ 50 mM, MgCl_2_ 250 mM, EDTA 250 µM and 2.5 mM, CaCl_2_ 500 mM, Glucose 2%, KH_2_PO_4_ 125 mM, CaCl_2_ 250 mM, Na-acetate 50 mM, Na-citrate 50 mM, (NH_4_)_2_HPO4 100 mM, (NH_4_)HSO_4_ 100 mM, and KH_2_PO_4_ 50 mM), Biofilm biomass was tested 7 days after the incubation, following previously described protocols [63,64]. All the experiments were performed in triplicates.

### 3.2. Microscopy

To characterize the biofilm formed by *Halomicronema* sp. CCY15110, a microscope slide, was placed transversely in a plastic container (SacO2 Microbox O95/114 + OD95, Belgium) with the cyanobacterial strain and 25 mL of BA+ culture medium. After 10 days, the strain formed a biofilm at the bottom of the container and on the slide, which could be used for imaging. Images were taken using the AXIO fluorescence microscope Imager Z2 (Zeiss, Jena, Germany) by staining cells with SYTO-9 (Thermo Fischer Scientific Inc., Waltham, MA, USA) and by employing the environmental scanning electron microscope (ESEM) FEG2500 FEI (FEI Europe, Eindhoven, The Netherlands) operating in low vacuum mode (60 Pa) with LFD (large field detector) to enable optimal imaging of electron secondary electrons (SEs). The pressure-limited conical aperture (PLA) of 500 μm improved the signal available for the QUANTAX XFlash X-ray detector (Bruker, Billerica, MA, USA). SE imaging was performed at 10 keV with a beam size of 2.5 μm [65].

### 3.3. Total Phenolic Content, DPPH, and ABTS Assays

Lyophilized extracts were tested following published protocols to determine the total phenolic compounds [66,67], and the antioxidant profile by performing the DPPH inhibition assay [66,68] and the ABTS assay [31]. The experiments were performed in triplicates.

### 3.4. Gene Selection and Primer Design

To design the specific primers to study specific gene expression in cyanobacteria, the list of the genes involved in the last steps of EPS synthesis was first retrieved from the literature [10,17,19]. The search was focused on the EPS-related systems known to be conserved across species (Wzy transporter-dependent pathway, ABC transporter-dependent pathway, and the synthase-dependent pathway) along with the ones involved in lipopolysaccharide synthesis. Having identified from the literature all the members involved in the different systems, their presence within the genomes of *Halomicronema* sp. was checked using NCBI and the txid 2767773 [23] (https://www.ncbi.nlm.nih.gov/bioproject/?term=PRJNA658956, accessed on 10 July 2023). When a match was found, the gene sequence was retrieved, and specific primers were designed using NCBI Primer Blast (https://www.ncbi.nlm.nih.gov/tools/primer-blast/index.cgi, accessed on 20 July 2023) with the following specification: 60 °C optimal melting temperature, and amplicon size between 120 and 170 bp. Similarly, the primer design was also performed on the housekeeping gene *secA*. For a complete list, see Table S4.

### 3.5. Gene Expression Analysis by RTq-PCR

Total RNA was isolated from cells growing for one week in BA+ (T 23 °C, 16 h light and 8 h dark). During the light condition, chemicals (MgSO_4_ 50 mM, EDTA 250 μM, NaCl 340 mM, NaCl 680 mM, and NaCl 1 M) were added and maintained for 2 h. One well was used as a control condition, and no chemicals were added. After 2 h of treatment, the cells were harvested for RNA extraction. RNA was recovered from one well using the RNeasy^®^ Mini Kit (Qiagen GmbH, Hilden, Germany) according to the manufacturer’s instructions. RNA concentrations were determined using a spectrophotometer VARIAN Cary 50 UV-VIS (Agilent Technologies, Santa Clara, CA, USA). Total RNA (500 ng) was reverse transcribed into cDNA using a OneScript ^®^ cDNA Synthesis Kit (which also contains AccuRT Genomic DNA Removal Kit purchased from Applied Biological Materials Inc., Richmond, BC, Canada) according to manufacturer instructions. The subsequent RTq-PCR was based on 20 ng of cDNA template, PowerUp SYBR Green Master Mix (ThermoFisher Scientific, Wilmington, NC, USA), with 250 nM of each forward and reverse primer. The reaction was conducted in the CFX96 Touch Real-Time PCR Detection System (Bio-Rad, Hercules, CA, USA), with the following program: 95° C for 5 min, followed by 40 cycles at 95 °C for 15 s, 60 °C for 60 s, immediately followed by melting curve analysis. The data were analyzed with the 2^−ΔΔCt^ method as described [69] using *secA* as a housekeeping gene as it had stable expression in all the conditions tested. The experiment was performed with three biological replicates.

### 3.6. RNA Sequencing

Total RNA was isolated from cells using RNAprotect^®^ Bacteria Reagent (Qiagen GmbH, Hilden, Germany), RNeasy^®^ Mini Kit (Qiagen GmbH, Hilden, Germany), and DNase I RNase-free (ThermoFisher Scientific, Wilmington, NC, USA). After extraction, the concentration and purity of RNA were determined using the NanoDrop 2000 (ThermoFisher Scientific, Wilmington, NC, USA). The integrity of the RNA was assessed by gel electrophoresis on 1.5% agarose gel.

To perform RNA sequencing, three total RNA samples were sent to the GALSEQ S.r.l. Company (Milan, Italy). The concentration and purity were measured with a spectrophotometer, and the quantitative assessment of RNA integrity (RIN) was determined with a 4200 TapeStation system (RNA ScreenTape Assay kit) (Agilent Technologies, Santa Clara, CA, USA). RNA was processed using NEBNext^®^ rRNA Depletion Kit (Bacteria) following the NEBNext^®^ Ultra™ II Directional RNA Library protocol Prep Kit for Illumina^®^ (New England Biolabs, Ipswich, MA, USA). RNA libraries were generated from 500 ng of total RNA, quantified and checked qualitatively with a 4200 TapeStation system (High Sensitivity D1000 kit), and then sequenced on an Illumina NovaSeq platform with paired-end reads 150 bp long and with an average depth of 20 million clusters per sample. The Fastq reads were checked with FastQC (https://www.bioinformatics.babraham.ac.uk/projects/fastqc/, accessed on 10 October 2022) to verify the overall quality of the reads and the presence of adaptor sequences. The paired reads were subsequently aligned to the genome of *Halomicronema* sp. CCY15110 (Assembly: GCF_016888165.1) using splice-aware aligner STAR v 2.7.9a [70] and indexed using Samtool [71]. Per-gene read counts were generated using the STAR quantMode geneCounts option [60].

### 3.7. Statistical Analysis

Data analysis was performed using SPSS (Ver.27, IBM Corp., Armonk, NY, USA). The Student’s *t*-test was performed to compare the effects of the different chemicals (see legend to Figure 2 for details).

## 4. Conclusions

This work represents an initial characterization of the cyanobacterium *Halomicronema* sp. in the field of biofilm formation and the causes that affect it. The expression of genes related to the production of EPS and transcription factors potentially involved in biofilm formation under different growth conditions were studied. This work identified possible biomarkers useful for *Halomicronema* sp. biofilm formation, including the EPS related genes *wza*1, *wzt*, along with *luxR*-05665, and some genes belonging to the TetR/AcrR and LysR families, as their expression is strongly sensitive to the growth conditions analyzed. In addition, given its antioxidant properties, as also observed in the case of *Spirulina* spp. it would also be useful to further study its antioxidant capacity. Of relevance was also the demonstration that *Halomicronema* sp can survive and grow under several stress conditions, including high salt. Therefore, an interesting application will be the possibility of testing if this bacterium has “Plant Growth Promoting” capacities, which can support crop growth in arable lands with high salinity.

## Figures and Tables

**Table 1:** Total phenolic concentration and antioxidant profile. The data are the mean of three independent experiments, and the standard deviation (SD) is reported. PI is the Percentage of Inhibition.

ASSAY	Mean ± SD
Total Phenolic Compounds (mg GAE g^−1^ extract DW)	0.7 ± 0.1
DPPH Inhibition (PI%)	63 ± 5
ABTS assay (PI%)	52 ± 4

**Table 2:** List of the transcription factors considered and their biological role.

Transcription Factor Family	Role of the Transcription Factor Family	Protein Name	Gene_ID	Average Value *	Protein_ID
**AbrB** family transcriptional regulator	Essential for cell survival, spore formation, and biofilm development [51]	AbrB-06010	JUK32_RS06010	5239	WP_204137943.1
**YebC/PmpR** family DNA-binding transcriptional regulator	Probably involved in regulation of quorum sensing, and virulence, regulates EPS and LPS production to form biofilm [52]	YebC-12275	JUK32_RS12275	283	WP_204139153.1
**TetR/AcrR** family transcriptional regulator	Regulation of essential processes (metabolism virulence, resistance, and biofilm formation) [53,54]	TetR-01335	JUK32_RS01335	768	WP_204137057.1
		TetR-14575	JUK32_RS14575	167	WP_204139591.1
		TetR-16580	JUK32_RS16580	1471	WP_204139976.1
		TetR-19750	JUK32_RS19750	517	WP_204140582.1
		TetR-23190	JUK32_RS23190	271	WP_204141244.1
		TetR-11335	JUK32_RS11335	702	WP_239112565.1
		TetR-13695	JUK32_RS13695	483	WP_239112647.1
**LuxR** C-terminal-related transcriptional regulator	Involved in quorum sensing, regulates virulence gene production, motility, plasmid transfer, and biofilm formation [55,56]	LuxR-02440	JUK32_RS02440	445	WP_204137261.1
		LuxR-04290	JUK32_RS04290	935	WP_204137618.1
		LuxR-05665	JUK32_RS05665	653	WP_239112365.1
**LysR** family transcriptional regulator	Regulates virulence, metabolism, quorum sensing, and motility [57]	LysR-07280	JUK32_RS07280	645	WP_204138189.1
		LysR-08635	JUK32_RS08635	1034	WP_204138452.1
		LysR-09925	JUK32_RS09925	266	WP_204138702.1
		LysR-11580	JUK32_RS11580	654	WP_204139027.1
		LysR-12705	JUK32_RS12705	613	WP_204139229.1
		LysR-20905	JUK32_RS20905	1630	WP_204140807.1
**GntR** family transcriptional regulator	Regulates motility, glucose metabolism, and virulence [58]	GntR-03040	JUK32_RS03040	2277	WP_239112277.1
		GntR-24680	JUK32_RS24680	1551	WP_239113044.1
**Crp/Fnr family** **transcriptional regulator**	Regulates virulence, the enzymes that degrade aromatic rings, genes involved in nitrogen fixation and photosynthesis [59,60]	Crp-04910	JUK32_RS04910	4875	WP_204137735.1
		Crp-18565	JUK32_RS18565	8743	WP_204140370.1
		Crp-20005	JUK32_RS20005	592	WP_204140632.1
		Crp-24180	JUK32_RS24180	3763	WP_204141438.1
**XRE family** **transcriptional regulator**	Unknown function, probably involved in the expression of virulence genes [61]	XRE-06870	JUK32_RS06870	3544	WP_204138108.1
**BolA** family transcriptional regulator	Involved in stress response, cell growth, morphology and division, biofilm development, inhibit motility [62]	BolA-25875	JUK32_RS25875	2291	WP_204141766.1

* Average value of the reads measured in the RNA-seq experiment under BA+ growing conditions.

## Data Availability

The RNA sequencing data presented in this study are openly available in the NCBI database, BioProject, under accession number PRJNA1200597 (https://www.ncbi.nlm.nih.gov/bioproject/ PRJNA1200597, uploaded on 15 December 24).

## Supplementary Materials

The following supporting information can be downloaded at https://www.mdpi.com/article/10.3390/ijms26020673/s1.
