# Supplementary material for: Biofilm Formation, Modulation, and Transcriptomic Regulation Under Stress Conditions in Halomicronema sp
Source: Int J Mol Sci. 2025 Jan 15;26(2):673. doi: 10.3390/ijms26020673 (PMC11765580; doi:10.3390/ijms26020673)
Supplement: Supplementary file 1 [file ijms-26-00673-s001.zip › ijms-3410315-supplementary.pdf]

Biofilm Formation, Modulation and Transcriptomic Regulation under stress conditions in *Halomicronema* sp.

Marina Caldara , Henk Bolhuis , Marta Marmiroli and Nelson Marmiroli

Table S1: Data output of RNA sequencing experiment

DATA OUTPUT

	Halo1	Halo2	Halo3
Million of input reads	23,70	21,90	19,02
Mean quality score	35.91	35.97	36.05
Average input read length	292	292	293
Uniquely mapped reads number	17878851	16413683	14937878
Uniquely mapped reads %	76.92%	76.31%	79.96%

Table S2: list of the top 50 most expressed genes retrieved by RNAseq in the control conditions. Data are reported for each sample and the average of the three.

	Halo1	Halo2	Halo3	Average	Product
JUK32_RS16510	1275426	1288587	1287548	1283854	transfer-messenger RNA
JUK32_RS17400	426625	372258	292831	363905	iron uptake porin
JUK32_RS11985	176782	149151	115261	147065	PstS family phosphate ABC transporter substrate-binding protein
JUK32_RS16145	159057	137155	111121	135778	PstS family phosphate ABC transporter substrate-binding protein
JUK32_RS10280	149248	117668	120460	129125	23S ribosomal RNA
JUK32_RS19160	126151	113249	100283	113228	photosystem II chlorophyll-binding protein CP47
JUK32_RS27125	108099	102833	99180	103371	2Fe-2S iron-sulfur cluster-binding protein
JUK32_RS01280	112729	109675	78991	100465	photosystem II q(b) protein
JUK32_RS19205	95073	80414	68290	81259	ammonium transporter
JUK32_RS08165	89250	78757	67727	78578	fructose-bisphosphate aldolase class II
JUK32_RS13030	74013	85469	50886	70123	RNase P RNA component class A
JUK32_RS01420	74002	71375	56774	67384	photosystem I core protein PsaB
JUK32_RS02465	72032	63732	56351	64038	amino acid ABC transporter substrate-binding protein
JUK32_RS01415	71471	65328	53633	63477	photosystem I core protein PsaA
JUK32_RS01530	72067	65465	48088	61873	photosystem II reaction center protein CP43
JUK32_RS02390	67394	59289	56347	61010	elongation factor Tu
JUK32_RS05645	69177	63636	46861	59891	ATP-dependent zinc metalloprotease FtsH3
JUK32_RS18960	64136	56302	49975	56804	peptidoglycan DD-metalloendopeptidase family protein
JUK32_RS12975	63801	56148	50250	56733	phycocyanin subunit beta
JUK32_RS04960	69357	58596	41694	56549	Fe(3+) ABC transporter substrate-binding protein
JUK32_RS06780	63345	55204	49683	56077	prepilin-type N-terminal cleavage/methylation domain-containing protein
JUK32_RS04935	64948	57521	43849	55439	urea ABC transporter substrate-binding protein
JUK32_RS13690	53266	49443	48170	50293	DEAD/DEAH box helicase
JUK32_RS12400	52056	49611	45370	49012	photosystem II q(b) protein
JUK32_RS13950	51430	48239	46507	48725	DUF1257 domain-containing protein
JUK32_RS11705	52855	47857	43418	48043	ATP-dependent Clp protease ATP-binding subunit
JUK32_RS19215	44186	43686	42257	43376	photosystem I reaction center subunit II
JUK32_RS02525	51902	47566	29319	42929	NAD(P)H-quinone oxidoreductase subunit 4
JUK32_RS25390	46360	41742	37076	41726	phycobilisome rod-core linker polypeptide
JUK32_RS00555	46341	40995	36268	41201	F0F1 ATP synthase subunit beta
JUK32_RS14825	44056	41277	37221	40851	septal ring lytic transglycosylase RlpA family protein
JUK32_RS24605	45132	40943	35120	40398	RNA polymerase sigma factor RpoD
JUK32_RS27110	52321	40626	26415	39787	choice-of-anchor I family protein
JUK32_RS01100	42935	40644	28020	37200	photosystem II q(b) protein
JUK32_RS26145	40281	36288	32812	36460	RNA-binding protein
JUK32_RS09515	40205	35840	32933	36326	DNA-directed RNA polymerase subunit beta
JUK32_RS12980	40946	36398	31151	36165	phycocyanin subunit alpha
JUK32_RS10510	33344	31337	42103	35595	pentapeptide repeat-containing protein
JUK32_RS25395	38926	33541	31486	34651	allophycocyanin subunit alpha
JUK32_RS04365	40582	34121	27344	34016	DUF839 domain-containing protein
JUK32_RS24715	32837	30995	36491	33441	RNA-binding protein
JUK32_RS26410	36281	33822	29410	33171	NAD(P)H-quinone oxidoreductase subunit 5
JUK32_RS11990	39785	34332	25153	33090	phosphate ABC transporter permease subunit PstC
JUK32_RS02315	34459	32038	30721	32406	hormogonium polysaccharide biosynthesis protein HpsA
JUK32_RS00620	33554	32863	30654	32357	photosystem I iron-sulfur center protein PsaC
JUK32_RS00635	34799	31655	28396	31617	transketolase
JUK32_RS14880	34642	30368	25759	30256	cell division protein SepF
JUK32_RS06315	38216	32865	19186	30089	TonB-dependent hemoglobin/transferrin/lactoferrin family receptor
JUK32_RS15230	32780	28974	25180	28978	phycobilisome rod-core linker polypeptide
JUK32_RS10295	28898	24517	33033	28816	16S ribosomal RNA

Table S3: EPS genes identified in *Halomicronema* sp genome

Pathway	Proteins involved	Biological Role	Presence in genome *Halomicronema* sp. CCY15110
**Wzy-dependent**	**Wzx**	Transfers lipid-bound oligosaccharide repeat units to the periplasmic side of the plasma membrane	YES, 2 variants *wzx1* e *wzx2*
	**Wxy**	Polymerizes lipid-bound oligosaccharides	YES
	Wzc	Export polymer across the outer membrane, Wzc is a polysaccharide copolymerase protein (PCP)	-
	**Wza**	Export polymer across the outer membrane, Wza is an outer membrane polysaccharide export protein (OPX)	YES, 2 variants: *wza1* e *wza2*
	Wzb	Controls the phosphorylation of Wzc	-
**ABC transporter dependent**	KpsM (o WzM)	Transfer the polysaccharide that has been fully polymerized to the inner leaflet of the plasma membrane (together they form the ABC transporter complex)	-
	**KpsT (o Wzt)**	Transfer the polysaccharide that has been fully polymerized to the inner leaflet of the plasma membrane (together they form the ABC transporter complex)	YES
	KpsC, KpsS, KpsF e KpsU	They export EPS across the outer membrane	-
	KpsE e KpsD	KpsE is a CFP and KpsD is an OPX	-
**Synthase dependent**	Alg8	Produces alginate	-
	BcsA	Produces cellulose and binds cGMP	-
	Alg44	Binds cGMP on Alg8	-
	AlgI, AlgF e AlgG	Modifies polymer in the periplasm	-
	AlgL	Degrades accumulated alginate	-

	BcsZ e BcsB	Degrades accumulated cellulose	-
	BcsQ	Probably necessary for polar localization of the apparatus	-
	AlgK e AlgE	They export the alginate, AlgK is a scaffold protein and AlgE is a porin	-
	BcsC	Exports cellulose, It is a scaffold protein	-
	**ExoD**	It is involved in EPS production, but its role is still unclear	YES, 2 variants: *exod1* e *exod2*
**Export of lipopolysaccharides**	**Wzy**	Polymerizes polysaccharide units	YES
	**WzT**	ATP-binding protein to the lipopolysaccharide transport system	YES
	**LptC**	Inner membrane protein of the LptBFGC export complex	YES
	**LptB**	ATPase of the LptBFGC export complex	YES
	LptG e LptF	Permease of the LptBFGC export complex	-
	LptA	Molecular chaperone responsible for lipopolysaccharide transport through the periplasm	-
	LptD e LptE	Proteins that insert lipopolysaccharides into the outer membrane	-

Table S4: list of the primers used in the RTqPCR gene expression analysis

Gene	Primer	Sequence (5'->3')	Amplicon length (BP)
bolA-25875	Forward	AACGACTTAACTGGCGGAGG	136
	Reverse	CGTGGATGGCCTCACTAGAC	
abrB-06010	Forward	AAGGCTAGGGAGTGTGGCTA	153
	Reverse	ACGATAAGTCGGTTCACGCC	
yebC-12275	Forward	GCGGACTACACCATCGAACA	157
	Reverse	CGGCCATGTCAAAGTTAGCG	
tetR-01335	Forward	TGACCAAGGCGGAACAAACT	138
	Reverse	ACAAGGCTCCCCGTGTAATG	
tetR-14575	Forward	AAAACGTCGGCATTGATCG	163
	Reverse	CCGTCGCCTGTTTTTCTAC	
tetR-16580	Forward	ACGAGTATGGATCGCGTAGC	160
	Reverse	CCGGTGGCCCACTTAAGATT	
tetR-19750	Forward	GGGCCTATCAGCAGCAGTTT	159
	Reverse	ACAGACAATCCACTGCCTCG	
tetR-23190	Forward	CTCAGCACACGCTTCAGGTA	158
	Reverse	GGGGTCATCATCCGCTTGAA	
tetR-11335	Forward	TACGTCTGGAGATTTCGCGG	148
	Reverse	GGCTCGACACAGCTCTCTTT	
tetR-13695	Forward	CAACTGCAATTGCCTGACCC	156
	Reverse	CTGGGCCGTAATCATCCCAA	
luxR-02440	Forward	TTGGAAATCAGCAAGCGCAC	143
	Reverse	GGCAGCGTACAGCAATTCAC	
luxR-04290	Forward	AATTGACCGAGCTGAGGGTG	142
	Reverse	AGTCCAGCAATCCAGCCTTC	

luxR-05665	Forward	TGTCAGCCAGATTGTGCAGT	156
	Reverse	GAAGAGGCGATCAGCCGTAA	
lysR-07280	Forward	CCATGTATTGCCAGAGGCGA	145
	Reverse	CAAGAGCATTTCGGCCACAC	
lysR-08635	Forward	AGCAGGGCCAACTCAGAATC	144
	Reverse	TCGCCATGCGCTCTTGAATA	
lysR-09925	Forward	TCGCGATCAGCGGTGAATAA	123
	Reverse	ACACCGCTCATAAAAGGCCA	
lysR-11580	Forward	AGCCGCTGTATGAAGAACCC	141
	Reverse	GTACCAGACGCTGCATACCA	
lysR-12705	Forward	GCCGCTCTTACCCTCGATAC	156
	Reverse	CAGCATGTTGTGTGCGTGAT	
lysR-20905	Forward	TCGGCTGAAGATTTCGCAGT	147
	Reverse	AGCAAGACGACGCCTAACTC	
gntR-03040	Forward	CAGTTTCCCCCAGGGTATCG	145
	Reverse	GGACGTAGATTCCTGAGCCG	
gntR-24680	Forward	TCGCCAACCTCATCCAACAA	146
	Reverse	CCGTCCTTGATAGCCTTGCT	
crp-04910	Forward	CGAGTCCTGAAACCGTCGAA	141
	Reverse	CATGACGACGCACTTTCACC	
crp-18565	Forward	TGACGGAAGTGGAATCGTCG	146
	Reverse	GGCAACCAAAAGCAGCAGTT	
crp-20005	Forward	AATGTCGCTGTTTGATGCGG	130
	Reverse	GATGTTGATGGCAATGCCCG	
crp-24180	Forward	ACCCAACATCATCGGCGAAT	146
	Reverse	CCGCTGCACCAGGTATAACA	
xre-06870	Forward	ACTCTTGACCCCACCGTTTG	152
	Reverse	AATGCTTTGGCCGCTTCTTC	
wzx1	Forward	ATATGCGCTGGTGTTGGTCA	174
	Reverse	ACCCCGAACACGCATAAACT	
wzx2	Forward	ATCAGTATCATCGGCGCGTT	187
	Reverse	GTAGCCACGCCCAAATACCT	

wzy	Forward<br>Reverse	CACCAAGTTTTGCCCGTCTG<br>TAATCACCAGCAGGACAGCG	172
wza1	Forward<br>Reverse	TTGACCTGGGGATGCCTCTA<br>GAATTGCTGGTAGGCAAGCG	128
wza2	Forward<br>Reverse	TTGGCCGAGATCAAGCAACA<br>AGCCGTCGGAATCAAAACCT	116
wzt	Forward<br>Reverse	AACGCTATCATCACCCCACC<br>TGCAGTAACGTGCTTTTGCC	163
exod1	Forward<br>Reverse	CGCTCAATCGAAGTTTTCGCA<br>AACAACCCAATCACCAGGCT	126
exod2	Forward<br>Reverse	ATTCCGGCACCAGGCTATTC<br>CCGCTTTGACCACCTTTTGG	151
lptc	Forward<br>Reverse	GGTTCTTGGTGGGAAAACGC<br>GTCGGCGTCTACTTTCCACA	123
secA	Forward<br>Reverse	CTGTTGGGCGTTCAGGATCT<br>CCCGGTAAACTCGTCCACAA	147
ftsZ	Forward<br>Reverse	TAAACGGTGAGACTGTGCCC<br>GTTTTCGGTTGCGGTGTTCA	167
psbA	Forward<br>Reverse	GTCACCAGCACCGAAAATCG<br>AGGAGGAGCGGCGATAAATG	111
